# Oral Administration of MBG to Modulate Immune Responses and Suppress OVA-Sensitized Allergy in a Murine Model

**DOI:** 10.1155/2014/567427

**Published:** 2014-03-05

**Authors:** Yu-Sheng Wu, Sherwin Chen, William Wang, Chung-Lun Lu, Chi-Feng Liu, Shiu-Nan Chen

**Affiliations:** ^1^College of Life Sciences, National Taiwan University, No. 1, Sec. 4, Roosevelt Road, Da'an District, Taipei City 10617, Taiwan; ^2^Department of Research and Development, Super Beta Glucan Inc., Irvine, CA, USA; ^3^Graduate Institute of Integration of Traditional Chinese Medicine with Western Nursing, National Taipei University of Nursing and Health Sciences, Taipei 11031, Taiwan

## Abstract

Recently studies performed on mushroom isolated polysaccharides demonstrated that **β**-(1,3)-glucan may affect the balance of Th1/Th2 cell response. Using ovalbumin (OVA) as a hypersensitivity inducer, we evaluated the ability of mushroom beta-glucan (MBG) in modulating Th1/Th2 cell responses in B6 mice. As compared to the control group, administration of MBG resulted in an increase of phagocytic activities, Th1 cytokine productions, immunoglobulins including IgG2A and IgA, and a significant expression of the splenic surface markers including CD3, CD4, CD8, and F4/80. In contrast, administration of MBG has significantly suppressed IgE and IgG1 levels and Th2 cytokines including IL-4, IL-5, and IL-6. Histopathological observation of MBG-treated followed by OVA-treated mice showed less filtration of eosinophil in pulmonary tissue sections. Our data suggested that administration of MBG treatments alters the natural course of the IgE-mediated hypersensitivities. In this investigation, we realize the mushroom beta glucan alter the Th2 response toward the Th1 in the allergic, resulting in a reduction in IgE productions which played a substantive role in reducing the severity of IgE-mediated hypersensitivity.

## 1. Introduction

A rapid increase in the prevalence of allergies and other chronic inflammatory diseases worldwide has highlighted the need to develop an effective intervention [1, 2]. Currently, the medical management of patients with allergy-related diseases includes allergen avoidance, pharmacotherapy, and immunotherapy, of which allergen-specific immunotherapy (SIT) is typically recommended for patients whose allergic symptoms cannot be ameliorated by environmental control and medications; however, this type of treatment involves an increased risk of anaphylaxis and physiological side effects [3–5].

Previous studies have shown that mushroom extract containing *β*-(1-3)-glucan, such as lentinan, protects against IgE-mediated allergy in a murine model [6, 7]. This protective effect is mediated through the stimulation of monocytes, Nature Killer cell (NK cell), and dendritic cells, resulting in the amelioration of a skewed Th1/Th2 balance and inflammation [8].

The Th1 and Th2 polarization is built on cytokine patterns, which begins when the antigen-presenting cells (APC) interact with the naive T cells and polarize into type 1 and type 2 cells in response to the type of antigen encountered [9]. While Th1 and Th2 cells secrete different cytokines, the Th1 cells are reliant on interlukin-2 (IL-2), interferon-*γ* (IFN-*γ*), and tumor necrosis factor (TNF), which are involved in cell-mediated immunity against pathogens, and the Th2 cells are mostly dependent on interleukin-4 (IL-4) and interleukin-5 (IL-5), which stimulate the production of IgE antibodies and eosinophil responses, resulting in the allergic diseases [10, 11]. While an imbalanced Th1/Th2 immune response is linked to certain hypersensitivity disorders such as allergy, asthma, and hay fever [12], studies have suggested that using biological response modifier (BRM) to restore the balance between Th1 and Th2 immune response can be a potential treatment option for the IgE dependent hypersensitivity [13]. *Ganoderma lucidum (G. lucidum)* is a medicinal mushroom which has been widely used as a folk medicine in oriental countries such China and Japan for hundreds of years for the immunomodulating and antitumor effects. Many biological available substances, in particular polysaccharides, with immunity enhancement effects have been isolated from the extract of *G. lucidum *[14]. The present study was designed to evaluate the efficacy of orally administrating MBG, a polysaccharide isolated from the *G. lucidum* to suppress the onset of the OVA-sensitized allergy in a murine model.

## 2. Materials and Methods 

### 2.1. MBG and Dosage Determination for Animal Study

In this experiment, mycelium of *G. lucidum*, subcultured and maintained in sterile YM agar (0.02%), was used for the production of MBG. The manufacturing process was initiated by preparing a culture medium containing glucose, lactose, galactose, sucrose, mannose, and yeast extract. Mycelium of *G. lucidum *was then introduced into the sterile medium and cultured using a shaker incubator at temperatures ranging from 27 to 32°C for 3–5 weeks to achieve full polymerization of MBG in the culture system. Subsequently, MBG from cultured mycelia was homogenized and disrupted using high speed homogenizer and ultrasonic vibration. The MBG solution was then filtered and concentrated using a ceramic membrane to strip most of the residual small molecules in the solution. The concentrated MBG was dried by lyophilization and then grinded into the powdered form. The sample was demonstrated to contain approximately 95% carbohydrate, 1% fat, 1% protein, 2% ash, and 0.8% water. Using Megazyme (Ireland) mushroom and yeast Beta-Glucan kit, the crude extract was demonstrated to contain approximately 60–65% of MBG (MBG). The molecular weight of MBG was analyzed by high pressure liquid chromatographic (HPLC) using Shodex SUGAR KS series containing KS-G, KS-804, and KS-805 columns and detected using RI 2000 detector. Molecular weight was determined by referring to the standard cure using standard molecules including STDP-800 (molecular weight M.W. 8 × 10^5^), STDP-400 (M.W. 4 × 10^5^), STDP-200 (M.W. 2 × 10^5^), STDP-100 (M.W. 1 × 10^5^), and STDP-20 (M.W. 2 × 10^4^). MBG was also processed for analysis of its glycosyl linkage. The sample was premethylated, depolymerized, reduced, and acetylated. The resultant partially methylated alditol acetates (PMAAs) were then analyzed by gas chromatography-mass spectrometry (GC-MS) according to the procedures described by York et al. and Ciucanu and Kerek [15, 16].

Result from the HPLC analysis showed that MBG powder contained high molecular weight particles in the range of 9.6~298 kDa. The result of GC-MS analysis showed that MBG powder contained 2-; 4-; and 6-linked galactopyranosyl residues and 3-; 4-; 3,4-; 2,4-; 4,6-; and 3,4,6-linked glucopyranosyl residues. It is the first beta-glucan extracted from the edible mushroom that has been generally recognized as safe (GRAS) under the US Food and Drug Administration (USFDA) regulation. The dosages for MBG treatments are determined based on a predetermined positive control, which is the equivalence of 17 mg/kg/day (0.85 g/50 kg/day) in accordance with the dosage level established by United States Department of Agriculture (USDA), and low, medium, and high dosages used in the experiments were the equivalence of 0.5, 3, and 10 times of the positive control dosage. The MBG solution was prepared fresh daily by dissolving assigned dose levels with 100 mL of sterile water.

### 2.2. Mice

A total of 50 male C57 BL/6 (B6) mice (six weeks old) of approximately 25 g each were obtained from the Laboratory Animal Center, National Taiwan University College of Medicine, for the experiment. All study procedures were performed in accordance with protocol approved by the National Taiwan University Animal and Use Committee (NTUAUC). For the present study, animals were housed in the Animal Housing Facility of National Taiwan University, College of Life Science, using polycarbonate cages with paddy husk bedding in the animal room. The room temperature and relative humidity were maintained at 21 ± 2°C and 55 ± 20%, respectively, with a 12-hour light/dark cycle. The animals were allowed to acclimatize for a minimum of six days before the initiation of experiments.

### 2.3. Treatment

50 B6 mice were divided into five groups based on stratified randomization by using body weights taken before the initiation of treatments. Mice were administered orally (gavage) once daily (SID) with MBG at the dose level of 0 mg/kg/day (control group), 17 mg/kg/day (positive control group; PC), 8.5 mg/kg/day (low dose group; low), 51 mg/kg/day (medium dose group; med.), and 170 mg/kg/day (high dose group; high) for 42 consecutive days. Ovalbumin (OVA) (Sigma, St. Louis, MO, USA) was prepared by mixing with complete adjuvant (Sigma, St. Louis, MO, USA) and each mouse was injected intraperitoneally (IP) with 20 *μ*g of OVA and 4 mg complete adjuvant in a total volume of 1 mL in a tail base at the 36th, 40th, and 41st day during course of the MBG treatment. All treated animals were euthanized by vertebral dislocation at the 43rd day of experiment for analysis. The dosing solutions were prepared fresh daily while the control animals received sterile water.

### 2.4. Assessment of NK Cell-Mediated Cytotoxicity

Assessment of the NK cell-mediated cytotoxicity was determined by the live/dead cell ratio using cell-mediated cytotoxicity kit (Invitrogen). To prepare for the target cells used in this part of the experiment, YAC-1 cells acquired from the Bioresource Collection and Research Center in Hsinchu, Taiwan, were adjusted to 1 × 10^6^/mL in cell density, followed by staining with DiOC-18 at 37°C in 5% CO_2_ for 20 min and a PBS rinse, and suspended to 1 × 10^6^/mL in RPMI 1640 medium. For the assay of NK cell-mediated cytotoxicity, the effector (NK cell) and target cells (YAC-1) were mixed in ratios (effector : target) of 5 : 1, 10 : 1, and 20 : 1, followed by adding the propidium iodide (PI) staining solution to each mixture. Finally, the cell mixtures were incubated at 37°C in 5% CO_2_ for 2 h and analyzed with flow cytometer (CyFlow Counter, Partec, USA). Lysed (PI+ and DiOC-18+) and viable (DiOC-18+ and PI−) YAC-1 cells were identified by their dual- or single-positive staining.

### 2.5. Assessment of Phagocytic Activity

On the 43rd day after the administration of MBG treatments, all mice were euthanized by cervical dislocation and the monocytes/phagocytes were obtained by washing the peritoneal cavity with RPMI-1640 medium. After centrifugation (2000 rpm, 20 min, 4°C), the precipitated cells were suspended in RPMI-1640 medium containing 10% FBS. After cultivation, adherent cells were collected. Monocytes/phagocytes were adjusted to 1 × 10^6^ cells in the 200 *μ*L of RPMI-1640 medium per well in a 96-well plate and incubated at 37°C for 2 h in 5% CO_2_. Upon removal of the nonattached cells after incubation, 100 *μ*L pHrodo *E. coli* BioParticles Conjugate in RPMI was added, followed by incubating at 37°C for 2 h in 5% CO_2_. RAW 264.7 cell line (net positive control) purchased from Bioresource Collection and Research Center in Hsinchu, Taiwan, was used to establish a baseline phagocytic activity. The phagocytic activity was measured by pHrodo BioParticles Conjugates for Phagocytosis kit (Invitrogen) using the following formula:

(1) phagocytic  activity  (%) =(net  experimental  phagocytosisnet  positive  control  (RAW  264.7)  phagocytosis)  ×100%.

### 2.6. Assays for Cell Surface Markers

Splenocytes were collected and stained with FITC-conjugated rat anti-mouse monoclonal antibodies including CD3, CD4, CD8, CD22, and F4/80 (eBioscience, San Diego, CA). Using a flow cytometry (CyFlow Counter, Partec, USA), fluorescence intensity (FI) was recorded and calculated for the mean from different MBG dosage groups.

### 2.7. Assays for Cytokine Productions

Blood samples collected were allowed 3–6 h to clot, centrifuged (4,000 rpm, 10 min), and stored at −20°C until the analysis. Splenocytes acquired from the mice were adjusted to 1 × 10^4^ cells per well and placed into a 96-well plate, added 10 *μ*g of OVA, and incubated for 24 h at 37°C in 5% CO_2_. Levels of cytokines IFN-*γ*, IL-2, IL-4, TNF-*α*, and IL-5 were measured from splenic cell culture supernatant and mice blood samples using ADI system kit (Enzo Life Sciences Inc., USA) and Mouse IL-5 ELISA kit (Abfrontier System, South Korea) in Microplate Spectrophotometer (*μ*Quant, BioTec).

### 2.8. Analysis of Serum Antibody Productions

Mice IgA, IgG2a, IgG1, and IgE antibodies were measured from serum using an ELISA kit (eBioscience, San Diego, CA, USA) according to the indication of the manufacturer, and the detection protocol of anti-OVA related antibodies was followed by Thumbikat et al. [17].

### 2.9. Histopathological Observations

A histopathological comparison was performed on the pulmonary tissues from the group which received MBG treatment after ovalbumin sensitization and the control group which received the MBG treatment only. Using Giemsa's staining. The pulmonary tissues were fixed in neutralized buffered formalin for observation under the microscope. The shamed mice were with MBG at the dose level of 0 mg/kg/day (control group).

### 2.10. Statistical Analysis

One-way ANOVA followed by Duncan's test was used to evaluate the statistical significance of differences amongst groups. A *P* value less than 0.05 (*P* < 0.05) was considered to be of statistical significance. Results are presented as mean ± SD. Different letters represent a statistically significant difference between the groups (*P* < 0.05); that is, a was different from b, b was different from c, and so forth, while the double-letter group (ab) shows that there was not statistically significant difference between this particular group (ab) and group a or b (*P* > 0.05).

## 3. Results

### 3.1. Effect of MBG on NK Cell-Mediated Cytotoxicity

**Figure 1:**
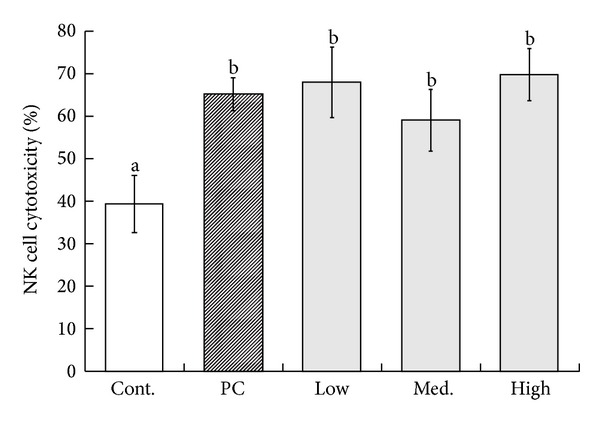
Detection of cytotoxic ability of splenic Nature Killer (NK) cells by measuring the percentage of dead Yac-1 cell line. Splenocyte samples were taken from experimental B6 mice. The mononuclear cell fraction was obtained from each sample. Each value represents the mean ± SD from ten independent experiments. Statistical significance was indicated by Duncan's test; the different letters represent a significant difference between the groups (*P* < 0.05). PC: positive control, low: low dose, med.: medium, high: high dose.

A statistically significant difference has been observed in the NK cell-mediated cytotoxicity between the control group and the groups receiving MBG treatments (*P* < 0.05). The NK cell cytotoxic observed for the control group was 39% (Figure 1), while the mean cytotoxicity for the positive control group was 67% (*P* < 0.05), 67.8% for the low dose group (*P* < 0.05), 59.2% for the medium dose group (*P* < 0.05), and 69.6% for the high dose group (*P* < 0.05), respectively. However, during the experiment, we were unable to establish a statistically significant dose response relationship in cytotoxic activities due to the fact that the medium dose group presented a lower cytotoxic activity than those of the low dose and positive control group.

### 3.2. Effects of MBG on the Phagocytic Activity

**Figure 2:**
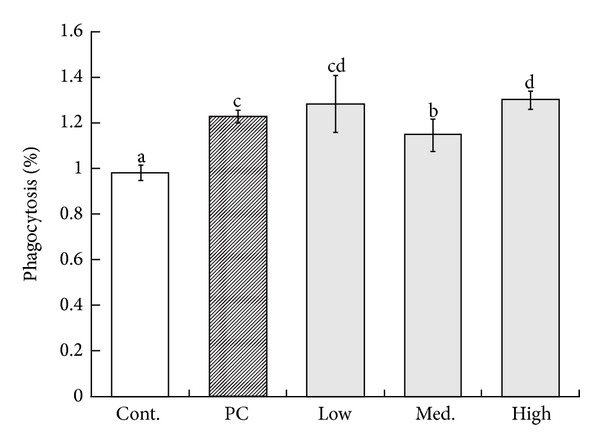
The comparison of the phagocytic activities performed in the splenocytes of the B6 mice administered with various MBG dosages. Each value represents the mean ± SD from ten independent experiments. Statistical significance was indicated by Duncan's test; the different letters represent a significant difference between the groups (*P* < 0.05). PC: positive control, low: low dose, med.: medium, high: high dose.

Phagocytotic activities observed in the MBG treatment groups were higher than the control group (*P* < 0.05). As shown in Figure 2, the mean of phagocytic activity for MBG-treated groups presented 123% in phagocytic activity for the positive control group (*P* < 0.05), 128% for the lower dose group (*P* < 0.05), 114% for medium dose group (*P* < 0.05), and 130% for high dose treated group (*P* < 0.05), respectively. However, no statistically significant trend has been observed in phagocytic activities with respect to the increasing dosage between different groups.

### 3.3. Expression of the Splenic Cell Surface Marker on MBG-Treated Animals

Results were presented in Table 1. The data indicated that groups which received MBG treatments in different dosages for consecutive 42 days have statistically significant increases in CD3, CD4, and CD8 expressions than those of the control group. However, no statistically significant difference has been observed in the CD22 expression in all groups. Moreover, we have observed a significantly higher F4/80 expression in the medium and high dosage groups when compared to the control group (*P* < 0.05). This suggested that MBG treatments at the medium or higher dosage could potentially induce F4/80 expression.

### 3.4. Effects on Cytokines and Serum Antibodies on MBG-Treated Animals

**Figure 3:**
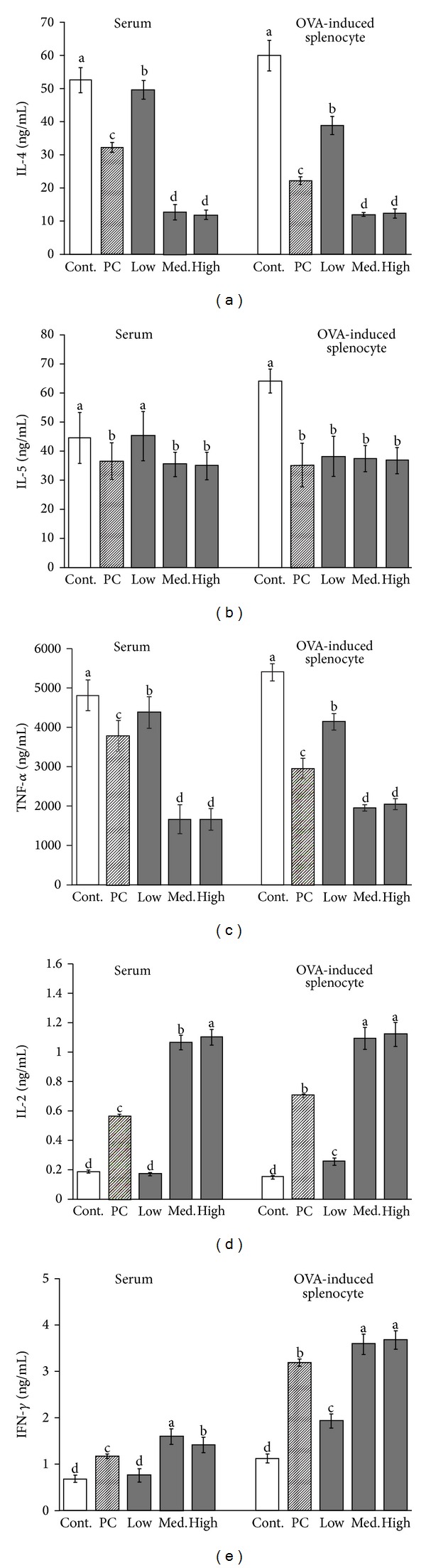
The comparison of cytokine concentrations (IL-4, IL-5, TNF-*α*, IL-2, and IFN-*γ*) in serum and splenocyte cultured fluid from the B6 mice administered with MBG in different dosages for 6 weeks. As shown in (a) and (c), concentrations of IL-4 and TNF-*α* were significantly decreased among MBG treatment groups. In contrary, concentrations of IL-2 and IFN-*γ* were significantly increased among MBG treatment groups ((d) and (e)). Each value represents the mean ± SD from ten independent experiments. Statistical significance was indicated by Duncan's test; the different letters represent a significant difference between the groups (*P* < 0.05). PC: positive control, low: low dose, med.: medium, high: high dose.

**Figure 4:**
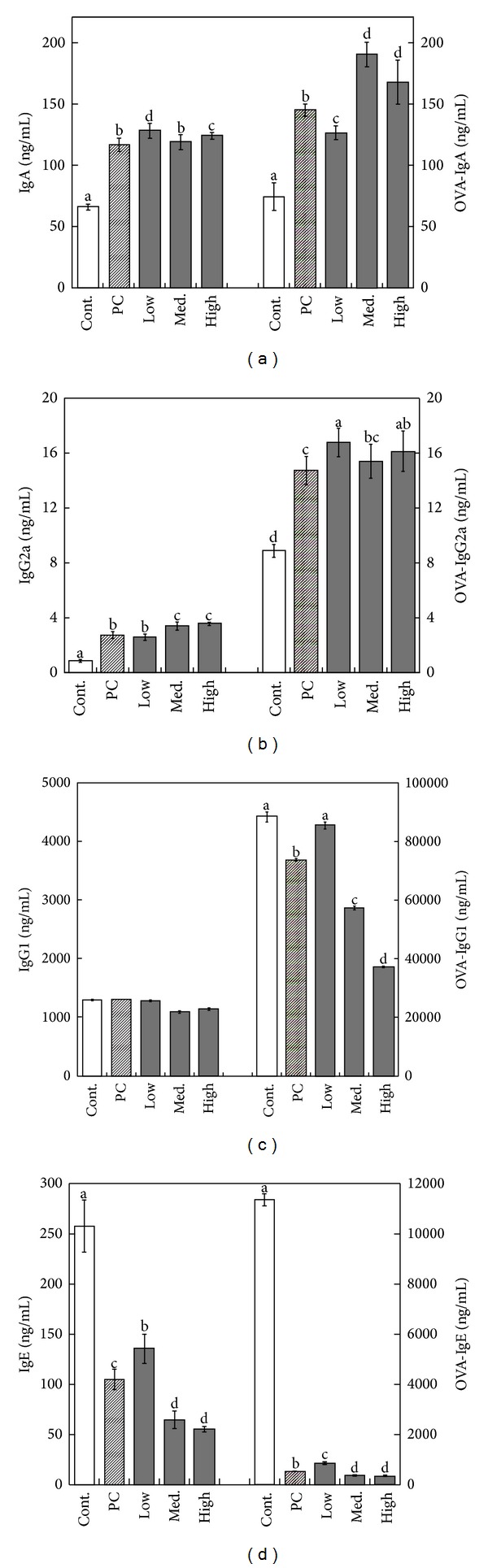
The comparison of IgA, IgG2a, IgG1, and IgE concentration in OVA-stimulated mice administered with MBG of different dosages. The productions of serum IgA and IgG2a were significantly elevated compared to the control group ((a) and (b)). The levels of IgG1 and IgE in OVA-stimulated group were significantly reduced in experimental groups receiving MBG when compared with the measurement from the control group ((c) and (d)). Each value represents the mean ± SD from ten independent experiments. Statistical significance was indicated by Duncan's test; the different letters represent a significant difference between the groups (*P* < 0.05). PC: positive control, low: low dose, med.: medium, high: high dose.

As illustrated in Figure 3, B6 mice that received the positive control, medium, and high dosage of MBG treatments showed statistically significant reductions in Th2 cytokines including IL-4, IL-5, and TNF-*α* in both OVA- and non-OVA-induced groups relative to the control group (*P* < 0.05) (Figures 3(a), 3(b), and 3(c)). In contrast, measurements of Th1 cytokines including IFN-*γ* and IL-2 from the same dosage groups showed statistically significant increments as compared to the control group (*P* < 0.05) (Figures 3(d) and 3(e)). Analysis from the serum immunoglobulins also indicated that IgA and IgG2a productions have increased significantly after administering MBG treatment for consecutive 42 days in both non-OVA- and OVA-induced groups (*P* < 0.05) (Figures 4(a) and 4(b)). In contrast, measurements of IgG1 and IgE have decreased significantly in both groups (*P* < 0.05) (Figures 4(c) and 4(d)).

### 3.5. Histopathological Observations

**Figure 5:**
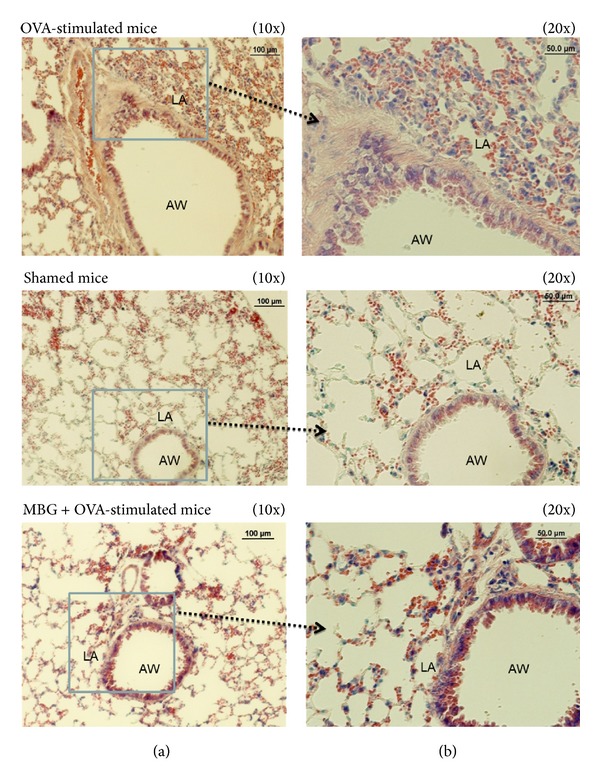
Histopathological observations of the pulmonary tissues obtained from three different experimental groups receiving OVA stimulation. Tissue sections at thickness of 5 *μ*m were made and stained with Giemsa for observation. Each figure was a representative from ten independent sections ((a) low-power magnification ×10 and (b) high-power magnification ×20). LA: lung alveolus. AW: airway. Shamed mice: the control animals receiving sterile water.

Histopathological findings confirmed that significant infiltration of the inflammation cells was observed when B6 mice were induced with OVA (Figure 5). Compared to the OVA-sensitized mice which received MBG treatments, a significant finding in eosinophil infiltration was observed in the group which received no MBG treatment. The result suggested that MBG treatment effectively suppresses the pulmonary inflammation by averting eosinophil infiltration in the pulmonary alveolus.

## 4. Discussion 

An increase in the prevalence of allergic diseases triggered by environmental allergens has been reported [2]. The pathogenesis of an allergic disease was initiated by the cross-linking of IgE molecules on the surface of the mast cells/basophils, resulting in the release of a host of mediators which ultimately cause hypersensitivity reactions [18, 19]. Polysaccharides isolated from mushrooms reveal a number of therapeutic [20] properties including immunomodulation [21] and anti-inflammation [22], which were mediated through the stimulation of immune cells such as NK cells, monocytes, dendritic cells, and T-lymphocytes [23–25]. Several studies also indicated that these bioactive compounds from the mushrooms prevent the progression of allergic diseases by promoting the cellular immunity and skewed the immunological function toward Th1 activity [26–28]. Findings from the previous studies suggested that a pretreatment of *G. lucidum* extracts increased the level of cytokine secretion in mice, as well as enhancing the activities of the cultured human blood-derived primary macrophages and NK cell-mediated cytotoxicity in vitro [29]. As demonstrated in the present study, we have observed a similar outcome in our experiment using MBG extracted from *G. lucidum*, where it enhanced the NK cell-mediated cytotoxicity of the treatment group by at least 30% at the effector/target cell ratio (E/T) of 20 : 1 as compared to the previous findings [30]. In the presented data, we also observed a similar enhanced percentage of the MBG-treated group compared to control group. Regarding the level of cytokine productions, as demonstrated from the results, the productions of Th2 cytokines such as IL-4 and IL-5 were significantly higher than those of the Th1 cytokines, including IL-2 and IFN-*γ* during an episode of IgE-mediated hypersensitivity. Additionally, an increase expression of cell surface markers including CD3, CD4, CD8, and F4/80 in mice treated with MBG, indicating the activation or proliferations of T helper cells, cytotoxic T cells (TC), macrophages, and monocytes prompted by MBG treatment. With the absence of CD22 expression, the result strongly suggested the variation in immunoglobulin profiles associated with cytokines produced from T cells instead of B cells. Furthermore, Levels of IL-4, IL-5, and TNF-*α* were significantly reduced after administering MBG treatments for 42 consecutive days. In contrast, measurements of IL-2 and IFN-*γ* were significantly increased. Therefore, we suggested that with daily MBG treatments drove the differentiation of T cell toward the Th1 cells rather than Th2 type. A similar result was reproduced from the groups receiving both OVA sensitization and MBG treatments, where the increased levels of Th1 immunoglobulins (IgA and IgG2a) and decreased levels of Th2 immunoglobulin (IgE and IgG1) have been observed. These findings strongly suggested that treatments of MBG modulate the immunoglobulin production by directing the naive T cells to differentiate towards the Th1 type. The results confirmed our hypothesis that MBG reduces the production of Th2 cells and increases the production of Th1 cells, which could potentially reduce the onset of hypersensitivity reactions. The Th2 cytokines secreted by bronchial epithelial cells, tissue mast cells, alveolar macrophages, and inflammatory cells were recognized as strong promoters for airway hyperresponsiveness [31, 32]. Eosinophils are responsible for the pathogenesis of hypersensitivity related inflammation [33]. The transmigration of eosinophils across the vascular and into the pulmonary tissues is a complex process triggered by Th2 cytokines such as IL-4, IL-5, and chemokines [34, 35]. As a result, prior to the histopathological analysis, we suggest that with a reduction in Th2 cytokine and immunoglobulin production levels results in a significant reduction of inflammations observed in the pulmonary tissue. B6 mice administered with MBG treatments showed significant reductions of eosinophil infiltration found in the pulmonary tissue. This result suggested a potential therapeutic approach by reducing the Th2 cells types to manage respiratory related hypersensitivities. Our results suggested that MBG could suppress IgE-mediated hypersensitivities by downregulating Th2 cytokine and immunoglobulin productions and upregulating those of Th1. The featured parameters of the Th1 immune response were identified as the reductions in serum IL-4, IL-5, IgG1, and IgE and the induction of IL-2, IgG2a, and IgA. Moreover, as only a few filtrations of eosinophil were identified from the pulmonary tissue sections during the histopathological observation, we have further identified a potential protective effect by MBG against type I hypersensitivity by shifting the Th1/Th2 balance toward Th1, and such pathways may play a substantive role in preventing and relieving symptoms associated with respiratory hypersensitivities.

## Figures and Tables

**Table 1:** The expression levels of surface markers on mice splenocytes from B6 mice treated with MBG in different dosages.

Surface markers	Control	Treatment mice			
		PC	Low	Med.	High
CD3 (%)	24.96 ± 3.3	28.01 ± 0.9*	27.94 ± 2.1*	28.32 ± 2.5*	28.46 ± 0.8*

CD4 (%)	16.36 ± 2.4	21.27 ± 0.9*	24.85 ± 4.4*	20.03 ± 1.9*	20.12 ± 1.5*

CD8 (%)	7.73 ± 1.8	8.81 ± 0.5*	9.38 ± 1.5*	8.33 ± 1.0*	9.19 ± 1.5*

CD22 (%)	52.95 ± 6.5	51.18 ± 0.7	50.19 ± 4.6	52.12 ± 4.9	51.42 ± 1.1

F4/80 (%)	91.00 ± 4.0	99.61 ± 0.1*	90.92 ± 1.7	99.74 ± 0.2*	99.59 ± 0.1*

*Significant difference at *P* < 0.05 level compared with those of control. The values shown in the flow cytometry profiles are the mean fluorescence intensity (MFI) indices. Each value represents the mean ± SD from ten independent experiments. Statistical significance is indicated by *P* values (Duncan's test).
